# Effects of Hospital Digitization on Clinical Outcomes and Patient Satisfaction: Nationwide Multiple Regression Analysis Across German Hospitals

**DOI:** 10.2196/40124

**Published:** 2022-11-10

**Authors:** Philip von Wedel, Christian Hagist, Jan-David Liebe, Moritz Esdar, Ursula Hübner, Christoph Pross

**Affiliations:** 1 Chair of Economic and Social Policy WHU - Otto Beisheim School of Management Vallendar Germany; 2 Department of Medical Management Faculty of Health Sciences Medical School Hamburg Hamburg Germany; 3 Health Informatics Research Group Faculty of Business Management and Social Sciences University of Applied Sciences Osnabrueck Osnabrueck Germany; 4 Department of Health Care Management Berlin University of Technology Berlin Germany

**Keywords:** health care information technology, electronic health records, hospital digitization, quality of care, clinical outcomes, patient satisfaction, user-perceived value

## Abstract

**Background:**

The adoption of health information technology (HIT) by health care providers is commonly believed to improve the quality of care. Policy makers in the United States and Germany follow this logic and deploy nationwide HIT adoption programs to fund hospital investments in digital technologies. However, scientific evidence for the beneficial effects of HIT on care quality at a national level remains mostly US based, is focused on electronic health records (EHRs), and rarely accounts for the quality of digitization from a hospital user perspective.

**Objective:**

This study aimed to examine the effects of digitization on clinical outcomes and patient experience in German hospitals. Hence, this study adds to the small stream of literature in this field outside the United States. It goes beyond assessing the effects of mere HIT adoption and also considers user-perceived HIT value. In addition, the impact of a variety of technologies beyond EHRs was examined.

**Methods:**

Multiple linear regression models were estimated using emergency care outcomes, elective care outcomes, and patient satisfaction as dependent variables. The adoption and user-perceived value of HIT represented key independent variables, and case volume, hospital size, ownership status, and teaching status were included as controls. Care outcomes were captured via risk-adjusted, observed-to-expected outcome ratios for patients who had stroke, myocardial infarction, or hip replacement. The German Patient Experience Questionnaire of Weisse Liste provided information on patient satisfaction. Information on the adoption and user-perceived value of 10 subdomains of HIT and EHRs was derived from the German 2020 Healthcare IT Report.

**Results:**

Statistical analysis was based on an overall sample of 383 German hospitals. The analyzed data set suggested no significant effect of HIT or EHR adoption on clinical outcomes or patient satisfaction. However, a higher user-perceived value or quality of the installed tools did improve outcomes. Emergency care outcomes benefited from user-friendly overall digitization (β=−.032; *P*=.04), which was especially driven by the user-friendliness of admission HIT (β=−.023; *P*=.07). Elective care outcomes were positively impacted by user-friendly EHR installations (β=−.138; *P*=.008). Similarly, the results suggested user-friendly, overall digitization to have a moderate positive effect on patient satisfaction (β=−.009; *P*=.01).

**Conclusions:**

The results of this study suggest that hospital digitization is not an end in itself. Policy makers and hospitals are well advised to not only focus on the mere adoption of digital technologies but also continuously work toward digitization that is perceived as valuable by physicians and nurses who rely on it every day. Furthermore, hospital digitization strategies should consider that the assumed benefits of single technologies are not realized across all care domains.

## Introduction

### The Promise and Policy-Based Promotion of Digital Health

For decades, digitization has been discussed as a promising answer to the various issues health care systems face today. A growing stream of research has revealed the positive effects of health information technology (HIT), specifically on the quality of health care providers, such as hospitals [1-4]. However, the adoption of promising HIT, such as electronic health records (EHRs), computerized clinical decision support systems, or telemedical tools, continues to lag expectations, particularly in Western Europe [5-7]. In 2017, for example, almost 50% of German and Austrian hospitals lacked an EHR system entirely and relied on paper-based documentation [8,9]. Policy makers reacted and introduced comprehensive financial incentives for HIT adoption. In the United States, the 2008 Health Information Technology for Economic and Clinical Health Act provided >US $28 billion to health care providers for adopting EHR systems [10]. Through this program, >80% of the hospitals have installed an EHR system since 2015 [11]. In 2020, German policy makers announced the establishment of a Hospital Future Fund (Krankenhauszukunftsfonds), which provides up to €4.3 billion (US $4.24 billion) to hospitals for investments in digital infrastructure and emergency capacities [12]. Policy makers and hospital decision makers clearly state attaining clinical outcome improvements as a fundamental goal of these measures and investments [13]. However, what appears to be an intuitive relationship reveals uncertainties, especially for the quality effects of nationwide HIT programs.

### Existing Research on the Relationship Between Hospital Digitization and Quality

Several studies have examined the relationship between health care provider digitization and quality of care. Most research represents single-case studies in which the introduction of a certain type of HIT in a single institution has been investigated [1,2,14-21]. In most cases, HIT is associated with benefits to quality in terms of clinical outcomes or patient satisfaction. For example, HIT deployment supported timely pneumococcal vaccinations, improved guideline adherence related to antibiotic prescriptions, drove medication adherence among patients with diabetes, and improved patient satisfaction scores [14,22-24]. These studies represent a valuable indication of the effects of HIT on quality; however, most research covers the application of customized solutions in single organizations, and the potential existence of publication bias is frequently stated [1,21]. Hence, policy makers would benefit from additional information on whether these effects at the micro level also translate into nationwide hospital quality.

A smaller stream in the literature represents studies that analyze nationwide data on HIT adoption and quality [4,25-30]. For example, Jones et al [26] analyzed data from 2021 US hospitals and identified process quality improvements for patients with heart failure following the installation of basic EHRs. However, most of these studies exclusively focused on process quality in contrast to actual clinical outcomes or patient satisfaction. Furthermore, studies to date almost exclusively covered EHRs and the United States, which is likely because of the comparably early introduction of the Health Information Technology for Economic and Clinical Health Act. Policy makers in other geographical locations such as Europe could have reason to doubt the applicability of the results from these studies to their respective health care systems.

### Research Contribution of This Study

In summary, this study assessed the effect of the availability and user-perceived value of multiple digital HIT tools on clinical outcomes and patient satisfaction across German hospitals. Thereby, the authors attempted to comprehensively address several previously identified research gaps. First, the goal was to add to the small stream of nationwide HIT studies by analyzing a geographical location outside the United States, namely Germany. Other European countries are likely to follow Germany’s approach of introducing a HIT adoption program. Hence, information on whether the previously described findings from US-based studies translate to European health systems is of interest. Second, we attempted to capture quality in terms of patient satisfaction and actual clinical outcomes in contrast to process quality. The challenge related to clinical outcomes, such as mortality or surgical revisions, is that absolute values are prone to several confounding factors and impede comparability across hospitals. Relative outcome measures involving patient-specific risk adjustment resolve these issues and can be considered the gold standard in terms of clinical outcome metrics [31,32]. In addition, previous research gives reason to assume that the influence of hospital digitization on outcomes potentially differs between unpredictable emergency care and planned elective treatments [16,26,28]. Hence, this study captured quality in terms of risk-adjusted outcome measures and differentiated between elective and emergency care. Third, we attempted to capture the digital maturity of hospitals comprehensively in contrast to focusing on the mere adoption of single technologies. Today, several technologies beyond EHRs assist physicians and nurses from admission to discharge. In addition, research has identified human factors as the most significant barriers to the proper use of HIT [1,6,33]. Hence, it seemed reasonable to also consider the value of hospital digitization as perceived by everyday clinical users in the analysis. This study relied on a sophisticated digital maturity score capturing both the availability and user-perceived value of several HIT categories. Ultimately, the goal was to provide policy-relevant insights for the meaningful design of nationwide HIT incentivization programs. In addition, the study can also generate insights for hospitals pursuing the promise of clinical outcome improvements via digitization.

## Methods

### Measures for Hospital Digitization

This study relied on the 2020 Healthcare IT Report for data on digital maturity of German hospitals. The Healthcare IT Report (IT Report Gesundheitswesen) is a comprehensive survey of German, Swiss, and Austrian hospitals that was first executed in 2002 and is continuing with varying core themes until today. The 2020 version of the report that surveyed 492 German physicians and nurses in 2017 went far beyond the mere adoption of technologies [8]. The report structured hospital digitization around the workflow from admission to discharge and captured comprehensive information on the adoption and user-perceived value related to >50 subtechnologies. Furthermore, the survey methodology has been constantly scientifically validated [34,35]. The report captured the digital maturity of hospitals across 10 domains composed of several technologies. A subscore was determined for both the adoption and user-perceived values of the underlying technologies for each domain. Table 1 presents an overview of all domains and the maximum attainable subscores. The domain admission, for example, was composed of 3 underlying technologies, namely occupancy control, collection of medical history and patient information, and steering of emergency patients (triage). The underlying technologies of the other domains can be found in the report publication and Multimedia Appendix 1 [8]. Summing the adoption subscores of all domains ultimately resulted in an overall maturity in terms of *HIT availability*. Similarly, averaging the user-perceived value across all domains resulted in an overall maturity in terms of *HIT value*. The report also covered EHR installations’ availability and user-perceived value in a separate question. Hence, this is not captured in the scores illustrated in Table 1. EHR adoption was captured via a simple yes-or-no question, and the user-perceived value of EHRs was captured on a scale from 1 to 10.

**Table 1:** Overview of the subdomains of hospitals’ digital maturity captured by the 2020 Healthcare IT Report (including maximum attainable scores).

HIT^a^ domain	Maximum attainable scores	
	Adoption *HIT availability*	User-perceived value *HIT value*
Admission	30	10
Surgery preparation	30	10
Discharge	35	10
Clinical documentation	77	10
Order entry and reporting	42	10
Decision support	42	10
Patient safety	49	10
Supply functions	40	10
Interface functions	50	10
Telemedicine and monitoring	20	10
Total digital maturity	415	10

^a^HIT: health information technology.

### Measures for Clinical Outcomes

This study considered risk-adjusted clinical outcome measures provided in the Qualitätssicherung mit Routinedaten (QSR) data set for both elective and emergency care. At the hospital level, these measures took the form of observed-over-expected (O/E) ratios, with the value of expected incidents being risk adjusted. This implied a worse-than-expected performance at values >1. In Germany, a reliable source of risk-adjusted clinical outcomes is the hospital data of Germany’s largest statutory sickness fund, the Allgemeine Ortskrankenkasse (AOK). The internal research institute of the AOK, Wissenschaftliches Institut der AOK, is responsible for the central calculation of the risk-adjusted outcome data consolidated in the QSR database [36,37]. A consolidated O/E ratio for elective care was determined by averaging 4 risk-adjusted indicators related to hip replacement surgery due to coxarthrosis. The consolidated O/E ratio for emergency care was determined by averaging the 30-day risk-adjusted standardized mortality rates for patients who had stroke and myocardial infarction (heart attack), which are the most important and representative emergency care cases (Multimedia Appendix 2). Details on patient-based risk adjustments can be found in the indicator handbook for QSR data [38].

### Measures for Patient Satisfaction

This study relied on data from the Patient Experience Questionnaire (PEQ) to assess the relationship between HIT adoption or user-perceived value and patient satisfaction in German hospitals. In Germany, Weisse Liste collects data on patient satisfaction in cooperation with the statutory insurance funds AOK, Barmer, and Kaufmännische Krankenkasse (KKH) [39]. Weisse Liste relies on the PEQ, covering 15 questions sent to patients 2 to 8 weeks after hospital discharge [40]. The questions cover various factors from personnel friendliness to admission and discharge process satisfaction. The complete questionnaire is publicly available for review [41]. Experience is rated on a scale of 1 to 6, from best to worst.

### Model Development and Estimation

Since several outcome metrics were of interest, a multiequation framework, similar to previous research in this field, was used [32,42]. A 3-equation multiple linear regression model was formulated with emergency care outcomes, elective care outcomes, and patient satisfaction as dependent variables:

Emergency care outcomes = α_0_ + α_1_(HIT_adoption(t − 1)_) + α_2_(HIT_user_value(t − 1)_) + α_3_(#emergency cases) + α_4_(#emergency cases^2^) + α_5_(#total cases) + α_6_(Private ownership) + α_7_(Teaching status) + α_8_(#beds) + ε **(1)**

Elective care outcomes = α_0_ + α_1_(HIT_adoption(t − 1)_) + α_2_(HIT_user_value(t − 1)_) + α_3_(#elective cases) + α_4_(#total cases) + α_5_(Private ownership) + α_6_(Teaching status) + α_7_(#beds) + ε **(2)**

Patient satisfaction = α_0_ + α_1_(HIT_adoption(t − 1)_) + α_2_(HIT_user_value(t −1)_) + α_3_(#total cases) + α_4_(Geography) + α_5_(Private ownership) + α_6_(Teaching status) + α_7_(#beds) + ε **(3)**

In equations 1 and 2, which capture care outcomes, the dependent variables were represented by the consolidated O/E ratios based on the QSR database for emergency or elective care. Since this study deliberately differentiates between technology adoption and user-perceived value, both HIT*_adoption_* and HIT*_user_value_* measures were included as independent variables. Here, the total digital maturity score of the Healthcare IT Report (Table 1) was considered. Importantly, a time lag of 1 year (t − 1) was introduced. This implies that full technology operability and user education require time after the initial installment [33,43,44]. In addition, several other independent variables were included to control for hospital-level effects. First, the underlying number of cases related to clinical outcomes was included to capture the potential volume effects directly linked to emergency or elective case volumes. Since several studies proved a positive relationship between case volume and quality, this is a critical confounding factor to control for [45,46]. Second, the total number of inpatient cases, #total cases, was included to capture the overall busyness of the respective hospitals [47]. The hospital ownership status was considered via the independent dummy variable Private ownership, which controls for potential organizational effects [48]. Similarly, teaching effects were included by another independent dummy variable: teaching status [48,49]. Finally, the number of hospital beds, #beds, was considered to capture the possible size effects of hospitals [42,50]. Equations 1 and 2 differ only in the inclusion of *#related cases^2^* in equation 1. The existing research has indicated an inverse U–shaped relationship between case volumes and outcomes for emergency care [42]. By including both the regular and squared emergency case volumes, this effect could be captured if present in the underlying data.

In equation 3, the dependent variable patient satisfaction was represented by the average PEQ score across all questions. Independent variables remained mostly the same as in equation 2, except that potential volume effects were captured by considering only all inpatient cases, #total cases, of the hospital. Interestingly, controlling for geographic effects seems important when considering patient satisfaction, as research has shown higher average satisfaction in Eastern Germany [51].

The linear-in-parameter models were separately estimated by relying on ordinary least squares (OLS) regression analysis performed by the SAS JMP software. The HIT-related independent variables HIT *adoption (t − 1)* and HIT *user_value (t − 1)* were captured as 2017 values based on the 2020 Healthcare IT Report. Considering the previously mentioned 1-year lag of full operability and user education, all other variables were based on 2018 values. All dependent and independent variables capturing case volumes were measured in natural logarithms to account for unequal variation and ensure that the assumptions of OLS regressions were met. The robustness of all the estimated OLS regressions was tested to ensure that the coefficients were unbiased and close to the actual population values. This also included estimations of the regression models without control variables (Multimedia Appendix 3).

### Ethics Approval

First, because the present study does not involve human biological material, this research was exempt from ethics approval in accordance with the 1961 German Drug Law (BGBI. I S. 533) and the 1994 Medical Devices Act (BGBI. I S. 1963). Second, according to statutes of the ethics committees of the institutions of all authors, data analyses processing data which does not relate to identifiable living individuals, which is the case for this study, do not require ethical approval in line with national guidelines (Regulation [European Union] 2016/679 [General Data Protection Regulation]). This study was conducted in accordance with the 1964 Helsinki declaration and its later amendments or comparable ethical standards. All methods were carried out in accordance with relevant guidelines and regulations.

## Results

### Overall Study Sample

The initial 492 responses of the 2020 Healthcare IT Report were cleaned for significantly incomplete questionnaires and contradictory answers from 2 respondents of the same hospital. Moreover, only hospitals for which information on either clinical outcomes or patient satisfaction was available were included. The final study sample comprised a total of 383 hospitals. The study sample characteristics and sample frames corresponding to all 1925 German hospitals in 2018 are presented in Table 2.

**Table 2:** Sample characteristics (N=383).

Sample characteristics				Values		Sample frame^a^
**Structural**						
	**Ownership, n (%)**					
		Private	88 (22.9)		37%	
		Nonprivate	295 (77)		63%	
	**Teaching status, n (%)**					
		Teaching	199 (51.9)		51%	
		Nonteaching	184 (48)		49%	
	Beds, mean (SD)			319.72 (318.35)		258.8
	Total inpatient cases, mean (SD)			12,861.6 (14,194.0)		10,290.2
	**Geography, n (%)**					
		Eastern states	72 (18.7)		18%	
		Western states	311 (81.2)		82%	
**Digital maturity**						
	HIT^b^ adoption (2017; maximum 415), mean (SD)			183.1 (98.4)		N/A^c^
	HIT user-value (2017; maximum 10), mean (SD)			6.3 (1.7)		N/A
	**EHR^d^a doption (2017), n (%)**					
		Yes	126 (32.8)		N/A	
		No	118 (30.8)		N/A	
		Unsure	6 (1.5)		N/A	
		No information	133 (34.7)		N/A	
**Clinical outcomes**						
	**O/E^e^ ratio**					
		Emergency care^f^, mean (SD)	1.07 (0.48)		1.07	
		Elective care^g^, mean (SD)	1.05 (1.23)		1.07	
**Patient satisfaction**						
	Overall PEQ score^h^, mean (SD)			1.99 (0.31)		2.02

^a^Official 2018 hospital report [52], nationwide Qualitätssicherung mit Routinedaten data, and nationwide Patient Experience Questionnaire data.

^b^HIT: health information technology.

^c^N/A: not applicable.

^d^EHR: electronic health record.

^e^O/E: observed-over-expected.

^f^Value of 1 indicates as-expected outcome performance (ie, higher values indicate worse outcomes), n=267.

^g^Value of 1 indicates as-expected outcome performance (ie, higher values indicate worse outcomes), n=249.

^h^On a 1-to-6 scale from best to worst (ie, higher values indicate lower satisfaction), n=354.

The study sample of 383 hospitals represented approximately 20% of the 2018 sample frame (1925 hospitals). Table 2 indicates that the key descriptive metrics of the sample are very much in line with the broader German hospital landscape, that is, the sample frame, hence supporting sample representativeness. With an average of 319.72 (SD 318.35) beds and 12,861.6 (SD 14,194.0) inpatient cases, the sample seemed to capture slightly larger hospitals than the 2018 German average. Overall, patients appeared to be relatively satisfied, with an average patient satisfaction score of 1.99 (SD 0.31) on a scale from 1 to 6 (best to worst). Considering digital maturity, hospitals still failed to adopt several technologies, resulting in a comparably mediocre average score of 183.1 (SD 89.4) out of the maximum attainable 415. Finally, 50% (126/250) of the hospitals that provided information on EHRs indicated adoption, which is largely in line with the existing research [8,9].

### HIT and EHR Effects on Clinical Outcomes

Several regressions were estimated based on the linear-in-parameter model in equations 1 and 2. The clinical outcome dependent variables were represented by the O/E ratios of emergency care and elective care. For both the dependent variables separately, the effects of the overall HIT adoption and user-perceived value, adoption of EHRs, and user-perceived EHR value were captured in 3 separate regressions. The adoption and user-perceived value of EHRs were captured in 2 separate regressions to allow for a higher subsample size for the regression-covering availability. Hospitals with <5 cases as the basis for the clinical outcomes O/E ratio calculations were excluded owing to high outcome variability.

For emergency care, the adoption of either HIT (*P*=.54) or EHRs (*P*=.11) did not significantly affect outcomes. However, the clinical user-perceived value of overall HIT did have a significant positive effect on the outcomes, which was mirrored by a decrease in the O/E ratio (β=–.032; *P*=.04). On the other hand, EHR adoption or user-perceived value did not influence the emergency outcomes. When looking at the control variables, model I indeed revealed an inverted U–shaped relationship between emergency case volumes and outcomes. This was represented by a positive influence of regular volumes (β=–.559; *P*<.001) and a negative influence of squared volumes (β=.058; *P*=.004). In addition, hospitals with higher overall inpatient case volumes generated worse emergency care outcomes (β=.102; *P*=.04; Table 3).

**Table 3:** Ordinary least squares (OLS) estimates for linear-in-parameter regressions capturing the impact of health information technology (HIT) and electronic health record (EHR) on emergency care clinical outcomes.

Dependent variable		ln^a^ (O/E ratio emergency care)^b^							
		Model I (HIT)			Model II (EHR adoption)			Model III (EHR user value)	
		β (SE)	*P* value	β (SE)		*P* value	β (SE)		*P* value
Intercept		.557 (0.506)	.27	.479 (0.65)		.46	−.563 (1.007)		.58
HIT adoption^c^		−.001 (0.001)	.54	N/A^d^		N/A	N/A		N/A
HIT user-value^e^		−.032 (0.016)	.04	N/A		N/A	N/A		N/A
EHR adoption^f^		N/A	N/A	.053 (0.034)		.11	N/A		N/A
EHR user-value^e^		N/A	N/A	N/A		N/A	−.018 (0.022)		.42
**#beds**									
	<150	.037 (0.087)	.67	−.082 (0.108)		.45	−.087 (0.147)		.55
	150-300	.027 (0.046)	.56	−.002 (0.061)		.97	.033 (0.077)		.66
	301-600	−.035 (0.049)	.47	−.002 (0.064)		.97	.065 (0.082)		.43
	>600	−.028 (0.086)	.74	.087 (0.114)		.45	−.01 (0.14)		.94
ln(#total cases)		.102 (0.051)	.04	.026 (0.068)		.70	.134 (0.086)		.12
ln(#emergency cases)		−.559 (0.167)	<.001	−.288 (0.214)		.18	−.203 (0.286)		.48
ln(#emergency cases)^^2g^		.058 (0.019)	.004	.027 (0.025)		.28	.018 (0.033)		.58
Teaching(yes)		−.036 (0.032)	.26	−.015 (0.214)		.72	−.022 (0.054)		.68
Private(yes)		.007 (0.037)	.83	.017 (0.046)		.72	.059 (0.060)		.33
Subsample size		261			174			82	
*R* ^2^		0.098			0.047			0.117	
*F* value		2.727	.003	0.905		.52	1.061		.40

^a^ln implies natural logarithm.

^b^O/E (observed-over-expected) ratio implies better performance with lower values.

^c^On a 0-to-415 scale from worst to best.

^d^N/A: not applicable.

^e^On a 1-to-10 scale from worst to best.

^f^Adoption of EHR.

^g^Tests for an inverse U–shaped relationship between case volumes and outcomes for emergency care.

Following an exploratory approach, additional regressions were estimated to better understand whether any subdomain (Table 1) had a prominent effect on the identified significant relationship at the overall HIT level. All HIT subdomains were separately regressed against emergency care outcomes. The admission subdomain generated statistically significant results (Multimedia Appendix 3). The user-perceived value of admission HIT had significant effects on emergency care outcomes (β=–.023; *P*=.07). An inverted U–shaped relationship between emergency case volumes and outcomes was also identified in this regression.

For elective care, the analysis of the effects of HIT and EHR on outcomes revealed not only similarities to but also differences from emergency care. In line with the emergency care results, the adoption of either HIT (*P*=.54) or EHRs (*P*=.84) did not significantly affect the elective care outcomes (Table 4). Although a higher user-perceived value of overall HIT did not affect outcomes either, clinical users’ satisfaction with EHRs had a significant effect, as shown in model III (β=–.138; *P*=.008). Of the control variables included in the regression, the number of elective care cases showed significant effects in models I and III, indicating a positive relationship between case volume and outcome quality in the elective care field. Interestingly, hospital size was a significant factor, with smaller hospitals performing better than larger ones.

**Table 4:** Ordinary least squares (OLS) estimates for linear-in-parameter regressions capturing the impact of health information technology (HIT) and electronic health record (EHR) on elective care clinical outcomes.

Dependent variable		ln^a^ (O/E ratio elective care)^b^							
		Model I (HIT)			Model II (EHR adoption)			Model III (EHR user value)	
		β (SE)	*P* value	β (SE)		*P* value	β (SE)		*P* value
Intercept		3.005 (1.495)	.046	3.4 (2.07)		.10	2.973 (2.741)		.28
HIT adoption^c^		.001 (0.001)	.54	N/A^d^		N/A	N/A		N/A
HIT user-value^e^		.038 (0.034)	.26	N/A		N/A	N/A		N/A
EHR adoption^f^		N/A	N/A	−.013 (0.063)		.84	N/A		N/A
EHR user-value^e^		N/A	N/A	N/A		N/A	−.138 (0.05)		.008
**#beds**									
	<150	−.529 (0.199)	.009	−.716 (0.229)		.002	−.42 (0.329)		.21
	150-300	−.075 (0.099)	.45	−.071 (0.125)		.57	−.089 (0.189)		.64
	301-600	.415 (0.108)	<.001	.5 (0.123)		<.001	.393 (0.181)		.03
	>600	.189 (0.191)	.32	.287 (0.228)		.21	.116 (0.329)		.73
ln(#total cases)		−.239 (0.157)	.13	−.266 (0.211)		.21	−.132 (0.283)		.64
ln(#elective cases)		−.324 (0.064)	<.001	−.269 (0.079)		.001	−.278 (0.115)		.02
Teaching(yes)		−.059 (0.065)	.36	−.114 (0.078)		.15	−.03 (0.12)		.81
Private(yes)		−.054 (0.072)	.45	−.062 (0.082)		.45	−.173 (0.119)		.15
Subsample size		184			118			59	
*R* ^2^		0.237			0.265			0.375	
*F* value		5.997	<.001	4.915		<.001	3.753		.002

^a^ln implies natural logarithm.

^b^O/E (observed-over-expected) ratio implies better performance with lower values.

^c^On a 0-to-415 scale from worst to best.

^d^N/A: not applicable.

^e^On a 0-to-10 scale from worst to best.

^f^Adoption of EHR.

### HIT and EHR Effects on Patient Satisfaction

Several regressions were estimated based on the linear-in-parameter model in equation 3, where the PEQ scores represented patient satisfaction. To ensure representativeness, the estimation excluded patient satisfaction PEQ scores based on <20 respondents per hospital. An overview of all the estimated models and their respective subsample sizes are presented in Table 5. In line with the effects on clinical outcomes, mere technology adoption did not significantly affect the overall patient satisfaction, that is, the overall PEQ score (*P*=.41). However, the user-perceived value of HIT did moderately affect patient satisfaction (β=–.009; *P*=.01), as indicated by model I of Table 5. Models II and III, which examined the impact of EHRs, did not identify any significant EHR-related effects. Across all models, higher bed numbers negatively affected satisfaction. In addition, in line with previous research, patients visiting hospitals in the Eastern German federal states were, on average, more satisfied.

An additional exploratory regression explicitly examining the relationship between admission HIT and admission satisfaction was estimated (Multimedia Appendix 3). Interestingly, the adoption of more technology in the admission implied a decrease in patients’ admission satisfaction (β=.002; *P*=.03). However, by contrast, the user-perceived value of admission HIT and patients’ admission satisfaction were significantly positively related (β=–.009; *P*=.02).

**Table 5:** Ordinary least squares (OLS) estimates for linear-in-parameter regressions capturing the impact of health information technology (HIT) and electronic health record (EHR) on patient satisfaction.

Dependent variable			ln^a^ (overall PEQ score)^b^												
			Model I (HIT)					Model II (EHR adoption)					Model III (EHR user value)		
			β (SE)		*P* value		β (SE)			*P* value		β (SE)			*P* value
Intercept			.694 (0.098)		<.001		.658 (0.124)			<.001		.822 (0.172)			<.001
HIT adoption^c^			.001 (0.001)		.41		N/A^d^			N/A		N/A			N/A
HIT user-value^e^			−.009 (0.004)		.01		N/A			N/A		N/A			N/A
EHR adoption^f^			N/A		N/A		.001 (0.007)			.93		N/A			N/A
EHR user-value^e^			N/A		N/A		N/A			N/A		−.006 (0.005)			.24
**#beds**															
	<150	−.097 (0.017)		<.001		−.087 (0.02)			<.001		−.122 (0.034)			<.001	
	150-300	−.008 (0.009)		.42		−.019 (0.013)			.14		−.014 (0.018)			.44	
	301-600	.032 (0.011)		.005		.026 (0.014)			.07		.033 (0.021)			.12	
	>600	.072 (0.018)		<.001		.08 (0.022)			<.001		.104 (0.031)			.001	
ln(#total cases)			.002 (0.01)		.82		.002 (0.013)			.90		−.013 (0.019)			.50
Geography (east)^g^			−.031 (0.007)		<.001		−.029 (0.01)			.005		−.029 (0.015)			.05
Teaching(yes)			.003 (0.008)		.67		.002 (0.009)			.83		.001 (0.014)			.97
Private(yes)			.003 (0.007)		.69		−.001 (0.009)			.95		−.016 (0.015)			.30
Subsample size			310					203					93		
*R* ^2^			0.322					0.259					0.35		
*F* value			15.824		<.001		8.516			<.001		5.669			<.001

^a^ln implies natural logarithm.

^b^PEQ (Patient Experience Questionnaire) on a 1-to-6 scale from best to worst.

^c^On a 0-to-415 scale from worst to best.

^d^N/A: not applicable.

^e^On a 1-to-10 scale from worst to best.

^f^Adoption of EHR.

^g^Effect of a hospital being located in an Eastern German state.

## Discussion

### Principal Findings

The analysis of our data set suggested that the adoption of HIT alone does not have a significant effect on either clinical outcomes or patient satisfaction. However, the degree of HIT value as perceived by hospital users did affect both clinical outcomes and patient satisfaction. Hospital digitization appears to be about quality instead of mere quantity.

In the elective care field, higher user-friendliness of EHRs for hospital staff appears to result in better outcomes, whereas digitization in other areas appears to matter less. By contrast, emergency care outcomes benefit from user-friendly overall digitization. Admission HIT, deemed valuable by physicians and nurses, is the strongest driver in this field. These identified differences appear to confirm practical experiences and logic. Elective care, represented by elective hip replacement surgery, relies on a comprehensive compilation of patients’ medical histories to support patient-specific diagnosis and therapy decisions. A user-friendly EHR is at the center of this collection and preparation process, representing a single source of information for each patient. The results of this study suggested that physicians and nurses who can rely on easy-to-access EHRs also generated better elective surgery outcomes. When it comes to emergency care, outcomes were positively affected by higher user-perceived value of overall hospital digitization. This overall effect was especially driven by user-friendly admission technology. This implied that rapid decision-making shortly after the incident most likely determines the outcomes. Taking an exclusive look at the roughly 270,000 annual stroke cases in Germany and their average 6.8% 30-day mortality, our results indicated that clinical user-perceived HIT value has the potential to reduce deaths [53,54]. Specifically, a 1-point improvement in user-perceived HIT value may result in a 3.15% improvement in mortality, which would ultimately translate to approximately 580 avoided deaths within 30 days per year. At this point, a brief look at the control variables of equations 1 and 2 also revealed interesting insights. Clinical outcomes for elective care, that is, hip replacements, benefit from higher procedure volumes. This finding was very much in line with the existing research [45,46,55-57]. The same applied to emergency care outcomes, at least to some extent. Here, an inverted U–shaped relationship between case volume and outcomes was identified, implying that emergency departments and their care teams are potentially overburdened at some point. Our analysis also suggested that smaller hospitals tend to perform better on elective care outcomes.

A similar picture emerged when considering patient satisfaction. Our analysis suggests that the mere adoption of overall HIT does not have significant effects on patient satisfaction. Digitization that appears as value adding from a hospital user perspective has a moderate positive impact on patients’ experiences. There are several potential reasons for this. First, well-designed digital tools covering admission, clinical documentation, order entry, discharge, or even catering management help physicians and nurses effectively execute care. This could mean shorter waiting times, better informed treating doctors, and smooth patient discharge processes, all of which likely positively influence patient satisfaction. Second, however, one can also assume hospital staff that are content with their everyday work environment, which includes HIT, to approach patients in a less stressed and more personal manner. This can also ultimately influence patient satisfaction through interpersonal mechanisms. Finally, an interesting phenomenon related to patient satisfaction with admission was revealed. Results showed that higher levels of admission digitization, for example, via tools for patient education and collection of medical history, resulted in lower admission satisfaction. Research has repeatedly warned of the undesired effects that digitization has on the patient-physician relationship, considering the very personal elements of health care [58]. Fortunately, results also showed that a higher degree of hospital user-perceived value of admission digitization can work against this effect and improve patient satisfaction. Hence, in cases such as admission, mere digitization could even be detrimental to patient satisfaction if not executed in a user-friendly manner for the hospital staff. Looking at the control variables, patients appeared to be less satisfied in larger hospitals. It can only be hypothesized that this might be related to a more personal care approach in smaller institutions.

The results of this study also provided valuable insights for both policy makers and hospitals. The results of this study suggested that an exclusive focus on driving HIT adoption will likely not be sufficient to achieve improvements in care quality. For example, the 2020 German Hospital Future Act also covers the costs of training and user education. Furthermore, it introduced a mandatory yearly digital maturity survey among all hospitals that applied for funding. Considering the limited data on the digital maturity of hospitals in Germany, this represents a desirable approach. However, of the 209 final questions included in the questionnaire, only 6 questions capture aspects of user satisfaction [59]. The results of this study advise policy makers to focus on the value of digitization as perceived by everyday clinical users instead of incentivizing the mere adoption of advanced digital tools. From the hospital’s perspective, much of what was previously discussed applies. First, hospital decision makers are advised to consider the views of the ultimate clinical users in their procurement decisions. Second, hospitals should not consider digitization projects completed after mere adoption but emphasize ongoing user training. Third, digitization strategies of health care providers benefit from differentiated approaches based on the respective area of care.

### Limitations

The results of this study are subject to several potential limitations. First, this study had to rely on a somewhat limited data set on hospital digitization in Germany. Although the 2020 Healthcare IT Report captured initial answers for almost 500 hospitals, the exclusion of significantly incomplete questionnaires and contradictory answers of 2 respondents from the same hospital resulted in a final overall sample of 383 hospitals. Moreover, responses related to the adoption and user-perceived value of EHRs were even more limited. This ultimately resulted in very differently sized subsamples, potentially impairing the comparability of the estimated regressions. These limitations strongly reveal the need for a structured and periodic digital maturity assessment among hospitals. Fortunately, as previously described, the 2020 Hospital Future Act introduced this assessment in Germany. However, the collected data must also be available to the research community.

Second, the study relied on patient satisfaction data from the PEQ, which collects answers on a Likert-type scale. There is some controversy about whether Likert-type responses can be averaged when used in statistical analyses with no unanimous results. However, since the PEQ data used in this study were normally distributed, we followed the standard approach of using average values [60].

Third, clinical outcome data were provided by the QSR database of the statutory insurer AOK. Hence, only the clinical outcome data for patients insured by the AOK were captured. However, as the AOK is by far the largest statutory insurer in Germany, with approximately 35% market share, the representativeness of the data can be assumed. Moreover, this study focused on the outcomes for two emergency care cases, namely stroke and myocardial infarction, and one elective care indication, namely hip replacement. Although stroke and myocardial infarction indeed represent the most essential emergency care indications in Germany, the results of this study might not be fully applicable to other areas of elective care.

Besides these primary variables of interest, this study also relied on several control variables when estimating OLS regressions. Looking at the R-square values of regressions, including care outcomes as the dependent variable, a significant share of variance was seemingly not explained by the included independent variables. Hence, risk-adjusted care outcomes appear to be influenced by a much wider variety of factors that were not captured in this study. This seems reasonable since care represents a highly personalized process that is also related to activities outside the hospital. Nevertheless, several additional mediator or moderator variables that could potentially impact the dependent variables of interest were not included in the regressions. Hence, the results of this study can only suggest a causal effect that the availability and user-perceived value of HIT have on care quality. On a similar note, reverse causality cannot be entirely ruled out. This would imply that hospitals which perform better on outcomes and patient satisfaction also invest more in HIT. However, ultimately, this study included the most prominent control variables used in research when assessing the effects on clinical outcomes and satisfaction to counter these potential limitations [2,20,21,47].

### Conclusions

This study examined the effects of digitization on clinical outcomes and patient satisfaction across German hospitals. The analysis of our data set suggested that the adoption of HIT alone does not significantly influence either outcomes or patient satisfaction, whereas the value of these technologies as perceived by physicians and nurses does positively influence patient outcomes and satisfaction. However, the results also implied that it is essential to differentiate between care indications and HIT subtypes. Whereas emergency care outcomes significantly benefit from user-friendly admission HIT, elective care outcomes significantly benefit from user-friendly EHR installations. Besides clinical outcomes, the user-perceived value of HIT significantly influenced patient satisfaction. Hospital staff working with HIT that is value adding from their perspective can treat patients in a manner that enhances the overall patient satisfaction. Besides HIT-related effects, the results suggest a positive relationship between case volume and outcomes in the elective care field. For emergency care, an inverse U–shaped relationship between the volume and outcomes was identified. Furthermore, elective care outcomes and patient satisfaction benefit from smaller hospital sizes in terms of bed numbers. Policy makers attempting to improve care quality via HIT are advised to focus on the value of digitization as perceived by everyday clinical users instead of incentivizing, capturing, or even imposing the mere adoption of digital tools. Hospitals are well advised to consider the views of the ultimate clinical users in their procurement decisions and invest in continuous training. In conclusion, hospital digitization can improve both clinical outcomes and patient satisfaction but only if deemed to be value adding by the physicians and nurses who rely on it every day.

## Appendix

Multimedia Appendix 1 Overview of the 2020 health care IT report scoring model.

Multimedia Appendix 2 Overview of the single outcome indicators considered for the calculation of consolidated overexpected values for elective and emergency care.

Multimedia Appendix 3 Robustness tests and statistical analysis of additional exploratory ordinary least squares regressions.

## Abbreviations

AOK: Allgemeine Ortskrankenkasse
EHR: electronic health record
HIT: health information technology
KKH: Kaufmännische Krankenkasse
O/E: observed-over-expected
OLS: ordinary least squares
PEQ: Patient Experience Questionnaire
QSR: Qualitätssicherung mit Routinedaten
