# Supplementary material for: Effects of Hospital Digitization on Clinical Outcomes and Patient Satisfaction: Nationwide Multiple Regression Analysis Across German Hospitals
Source: J Med Internet Res. 2022 Nov 10;24(11):e40124. doi: 10.2196/40124 (PMC9693730; doi:10.2196/40124)
Supplement: Multimedia Appendix 1 [file jmir_v24i11e40124_app1.pdf]

Multimedia Appendix 1

2020 Healthcare IT Report Scoring Model

	**Maximum attainable scores**	
**HIT domain**	***Adoption*** *"HIT availability" (summed)*	***User-perceived value*** *"HIT value" (averaged)*
**Admission**	**30**	**10**
Occupancy control	10	10
Anamnesis	10	10
Emergency room triage	10	10
**Surgery preparation**	**30**	**10**
Scheduling	10	10
Room planning	10	10
Resource planning	10	10
**Discharge**	**35**	**10**
Nursing report	7	10
Digital dictation	7	10
Medication plan	7	10
Communication w outpatient sector	7	10
Patient portal	7	10
**Clinical documentation**	**77**	**10**
Surgery docu.	7	10
Medical basis docu.	7	10
Physician letter	7	10
Wound docu.	7	10
Medical report	7	10
Anesthesia docu.	7	10
Hygiene docu.	7	10
Nursing docu.	7	10
Special nursing docu.	7	10
Therapy docu.	7	10
Intensive care docu.	7	10
**Order entry and reporting**	**42**	**10**
Laboratory data	7	10
Radiology incl. images	7	10
Radiology excl. images	7	10
Other exams	7	10
Electrophysiological exams	7	10
Councils	7	10
**Decision support**	**42**	**10**
**Access to clinical databases**	7	10
**Alarms**	7	10
**Clinical reminders**	7	10
**Medical guidelines**	7	10
**Medication support**	7	10

**Decision support for diagnostics and therapy**	7	10
**Patient safety**	**49**	**10**
Laboratory assay ID	7	10
CIRS	7	10
Patient ID	7	10
Checklists	7	10
Medication order	7	10
Electronic medical administration record	7	10
Medication tracking	7	10
**Supply functions**	**40**	**10**
Apothecary	10	10
Food orders	10	10
Material management	10	10
Material management incl standards	10	10
**Interface functions**	**50**	**10**
Duty planning	10	10
ADT patient planning	10	10
Electronic archive	10	10
Outpatient patient management	10	10
Quality management	10	10
**Telemedicine & -monitoring**	**20**	**10**
Telemedicine	10	10
Telemonitoring	10	10
**Total digital maturity**	**415**	**10**
