# Supplementary material for: Effects of Hospital Digitization on Clinical Outcomes and Patient Satisfaction: Nationwide Multiple Regression Analysis Across German Hospitals
Source: J Med Internet Res. 2022 Nov 10;24(11):e40124. doi: 10.2196/40124 (PMC9693730; doi:10.2196/40124)
Supplement: Multimedia Appendix 2 [file jmir_v24i11e40124_app2.pdf]

Multimedia Appendix 2

Overview of single outcome indicators considered for calculation of consolidated O/E values for elective and emergency care.

Consolidated O/E ratio	Averaged input indicators for consolidated O/E ratio
**O/E ratio emergency care**	Stroke (ICD: I61, I63 & I64)<br>- 30-day risk-adjusted standardized mortality rate<br><br>Myocardial infarction (heart attack) (ICD: I21 & I22)<br>- 30-day risk-adjusted standardized mortality rate
**O/E ratio elective care**	Hip replacement surgery due to coxarthrosis (OPS: 5-820.0/8/9/x)<br>- 90-day risk-adjusted standardized mortality rate<br>- Risk-adjusted revision surgery within 365 days<br>- Risk-adjusted surgical complications within 90/365 days<br>- Risk-adjusted femoral fracture within 90 days
