# Supplementary material for: Effects of Hospital Digitization on Clinical Outcomes and Patient Satisfaction: Nationwide Multiple Regression Analysis Across German Hospitals
Source: J Med Internet Res. 2022 Nov 10;24(11):e40124. doi: 10.2196/40124 (PMC9693730; doi:10.2196/40124)
Supplement: Multimedia Appendix 3 [file jmir_v24i11e40124_app3.pdf]

Multimedia Appendix 3

Robustness analysis

The robustness of all estimated OLS regressions was tested to assure that coefficients were unbiased and close to the actual population values (see Multimedia Appendix 2 for details). Results of the Shapiro-Wilk test suggested that the null hypothesis of normally distributed residuals cannot be rejected. Heteroscedasticity was assessed by applying the White test, which generated negative results. Multicollinearity was tested by determining Variance Inflation Factors (VIF) for all estimated coefficients. Besides the VIFs for $(\#emergency\ cases)$ and $(\#emergency\ cases)^2$, none of these was higher than five and, hence, it can be assumed that the model was not significantly affected by multicollinearity. Additionally, R^2 was determined to capture the proportion of variance for the dependent variables explained by independent variables. F-tests were executed, determining the overall significance of regressions. All estimated regressions except for the ones involving EHR effects on emergency care outcomes successfully passed the test. Furthermore, all regressions were additionally estimated without considering control variables, which generated the same direction for significant main effects. The exclusion of control variables naturally resulted in lower R^2 and F values (see below).

Table 3 OLS estimates for linear-in-parameter regressions capturing the impact of HIT and EHR on emergency care clinical outcomes ***(without control variables)***

**Dependent variable:**	**ln(O/E ratio emergency care)**[a]					
**Model**	**I (HIT)**		**II (EHR adoption)**		**III (EHR user value)**	
	$\hat{\beta}$ (SE)	*P*-value	$\hat{\beta}$ (SE)	*P*-value	$\hat{\beta}$ (SE)	*P*-value
Intercept	0.244	.026	-0.048	.299	0.174	.249
$HIT_{adoption}$[b]	-001	.823				
$HIT_{user-value}$[c]	-0.036	.024				
$EHR_{adoption}$[d]			0.108	.198		
$EHR_{user-value}$[c]					-0.019	.355
Sub-sample size	261		174		82	
R^2	0.02		0.015		0.011	
F-value	2.682	0.07	2.754	.198	0.865	.355

Note: Rounded figures, ln implies natural logarithm; [a]O/E ratio implies better performance with lower values; [b]on a 0-415 scale from worst to best; [c]on a 1-10 scale from worst to best; [d]implies adoption of EHR

Table 4 OLS estimates for linear-in-parameter regressions capturing the impact of HIT and EHR on elective care clinical outcomes ***(without control variables)***

**Dependent variable:**	**ln(O/E ratio elective care)**[a]					
**Model**	**I (HIT)**		**II (EHR adoption)**		**III (EHR user value)**	
	$\hat{\beta}$ (SE)	*P*-value	$\hat{\beta}$ (SE)	*P*-value	*P*-value	*P*-value
Intercept	-0.194	.464	-0.059	.564	0.902	.012
$HIT_{adoption}$[b]	0.001	.626				
$HIT_{user-value}$[c]	0.008	.818				

$EHR_{adoption}$[d]			-0.023	.872		
$EHR_{user-value}$[c]					-0.146	.008
Sub-sample size	184		118		59	
R^2	0.002		0.001		0.118	
F-value	0.147	.864	0.026	.872	7.621	.008

Note: Rounded figures, ln implies natural logarithm; [a]O/E ratio implies better performance with lower values; [b]on a 0-415 scale from worst to best; [c]on a 1-10 scale from worst to best; [d]implies adoption of EHR

Table 5 OLS estimates for linear-in-parameter regressions capturing the impact of HIT and HER on patient satisfaction ***(without control variables)***

**Dependent variable:**	**ln(Overall PEQ score)**[a]					
**Model**	**I (HIT)**		**II (EHR adoption)**		**III (EHR user value)**	
	$\hat{\beta}$ (SE)	*P*-value	$\hat{\beta}$ (SE)	*P*-value	$\hat{\beta}$ (SE)	*P*-value
Intercept	0.728		0.671	<.001	0.693	<.001
$HIT_{adoption}$[b]	0.001	.002				
$HIT_{user-value}$[c]	-0.014	.001				
$EHR_{adoption}$[d]			0.024	.151		
$EHR_{user-value}$[c]					-0.001	.963
Sub-sample size	310		203		93	
R^2	0.061		0.01		<0.001	
F-value	10.038	<.001	2.082	.151	0.002	.963

Note: Rounded figures, ln implies natural logarithm; [a]on a 1-6 scale from best to worst; [b]on a 0-415 scale from worst to best; [c]on a 1-10 scale from worst to best; [d]implies adoption of EHR

Overview of additional exploratory regressions

Table 1 OLS estimates for linear-in-parameter regressions capturing the impact of admission-HIT on emergency care clinical outcomes

**Dependent variable:**	**ln(O/E ratio emergency care)**[a]		
**Model**	**I (HIT)**		
	$\hat{\beta}$	SE	*P*-value
Intercept	0.887	0.553	.109
$Admission_{adoption}$[b]	0.001	0.004	.832
$Admission_{user-value}$[c]	-0.023	0.013	.073
#beds			
<150	0.066	0.096	.495
150-300	0.012	0.047	.799
301-600	-0.031	0.053	.552
>600	-0.046	0.091	.612
ln(#total cases)	0.083	0.174	.121
ln(#emergency cases)	-0.68	0.174	.001
ln(#emergency cases)^2 [d]	0.072	0.02	.001
Teaching[YES]	-0.02	0.035	.565
Private[YES]	0.007	0.039	.854
Sub-sample size	232		
R^2	0.113		
F-value	2.824		.003

Note: Rounded figures, ln implies natural logarithm; [a]O/E ratio implies better performance with lower values; [b]on a 0-30 scale from worst to best; [c]on a 1-10 scale from worst to best; [d]tests for an inversed U-shaped relationship between case volumes and outcomes for emergency care

Table 2 OLS estimates for linear-in-parameter regressions capturing the impact of admission-HIT on patient satisfaction with admission

**Dependent variable:**	**ln(Admission PEQ score)**[a]		
**Model**	**I (Admission-HIT)**		
	$\hat{\beta}$	SE	*P*-value
Intercept	0.073	0.121	.546
$Admission_{adoption}$[b]	0.002	0.001	.031
$Admission_{user-value}$[c]	-0.009	0.004	.015
#beds			
<150	-0.091	0.021	<.001
150-300	0.008	0.012	.527
301-600	0.022	0.014	.101
>600	0.061	0.021	.005
ln(#total cases)	0.061	0.013	<.001

Geography[East][d]	-0.029	0.009	.001
Teaching[YES]	-0.003	0.009	.763
Private[YES]	0.003	0.009	.775
Sub-sample size	267		
R^2	0.489		
F-value	27.29		<.001

Note: Rounded figures, ln implies natural logarithm; [a]on a 1-6 scale from best to worst; [b]on a 0-30 scale from worst to best; [c]on a 1-10 scale from worst to best; [d]Effect of hospital being located in an Eastern German state
